# Supplementary material for: Modelling Torsade de Pointes arrhythmias in vitro in 3D human iPS cell-engineered heart tissue
Source: Nat Commun. 2017 Oct 20;8:1078. doi: 10.1038/s41467-017-01125-y (PMC5715012; doi:10.1038/s41467-017-01125-y)
Supplement: Supplementary file 1 — Supplementary Information [file 41467_2017_1125_MOESM1_ESM.pdf]

Supplementary Information

Supplementary Figures and Figure Legends

Supplementary Figure 1

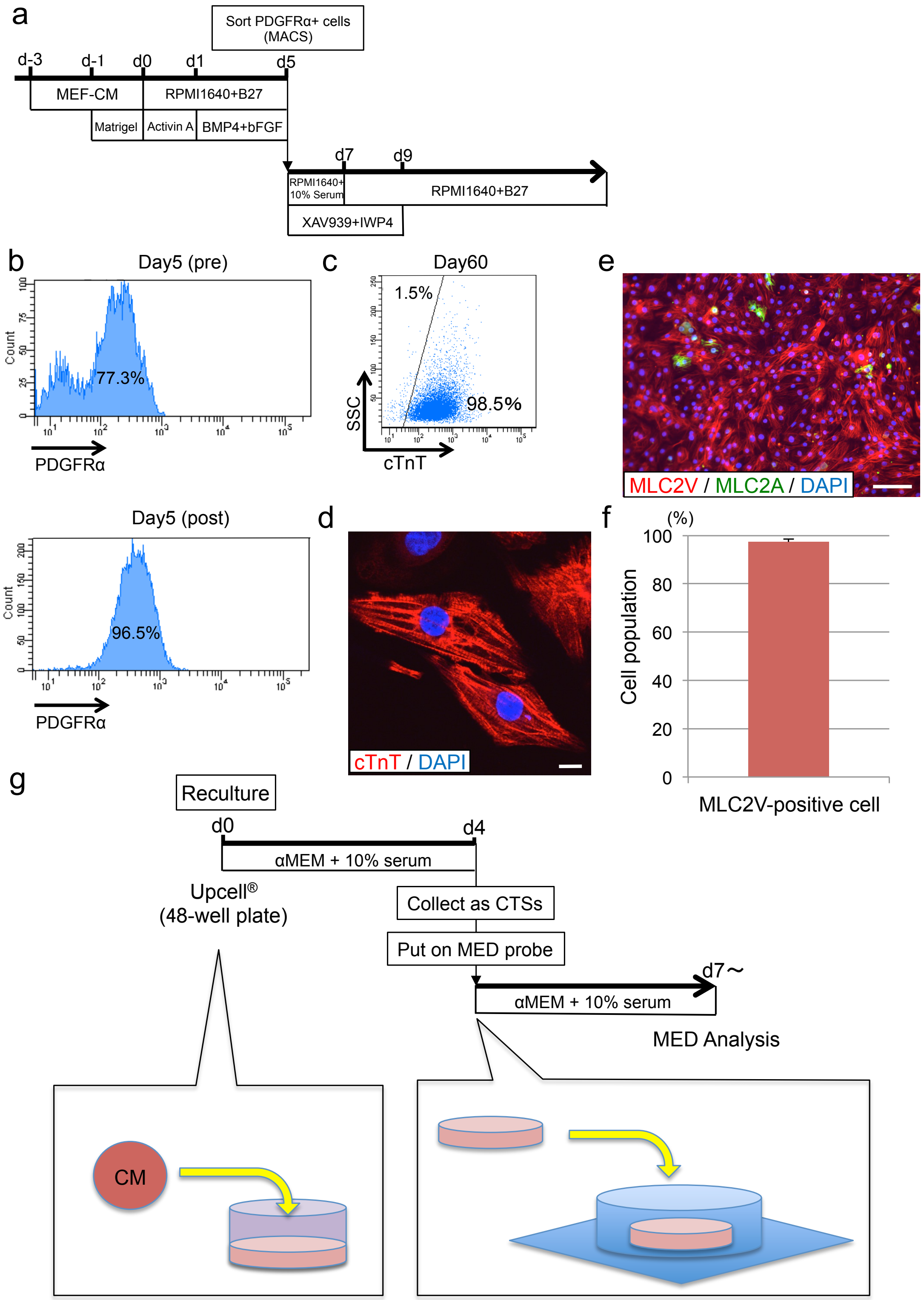

Supplementary Figure 1: Preparation of hiPSC-derived cardiomyocytes and 3D cell sheets with 100% cardiomyocytes.

(**a**) Schema for the cardiomyocyte differentiation protocol from hiPS cells. **(b)** Histograms of the flow cytometry analyses of day 5 PDGFRα-positive cell sorting with MACS. Representative histogram before and after sorting of PDGFRα-positive cells. **(c)** Representative flow cytometry dot plots for cTnT on differentiation day 60. **(d)** A confocal image with immunostaining for cTnT to identify cardiomyocytes (red) and DAPI to identify nuclei (blue). **(e)** Immunostaining for MLC2V and MLC2A in cardiomyocytes. **(f)** Prevalence of MLC2V-positive cells (±s.d., n=4). **(g)** Schema for the preparation of human iPS cell-derived 3D CTSs. ActA, activin A; αMEM, alpha minimum essential medium; bFGF, basic fibroblast growth factor; BMP4, bone morphogenetic protein 4; CM, cardiomyocytes; cTnT, cardiac troponin-T; DAPI, 4',6-diamidino-2-phenylindole; MACS, magnetic-activated cell sorter; MED: multi-electrode device; MEF-CM, mouse embryonic fibroblast conditioned medium; PDGFRα, platelet-derived growth factor receptor type alpha; SSC, side scatter. Scale bars: 10 μm in **d** and 100 μm in **e**.

Supplementary Figure 2

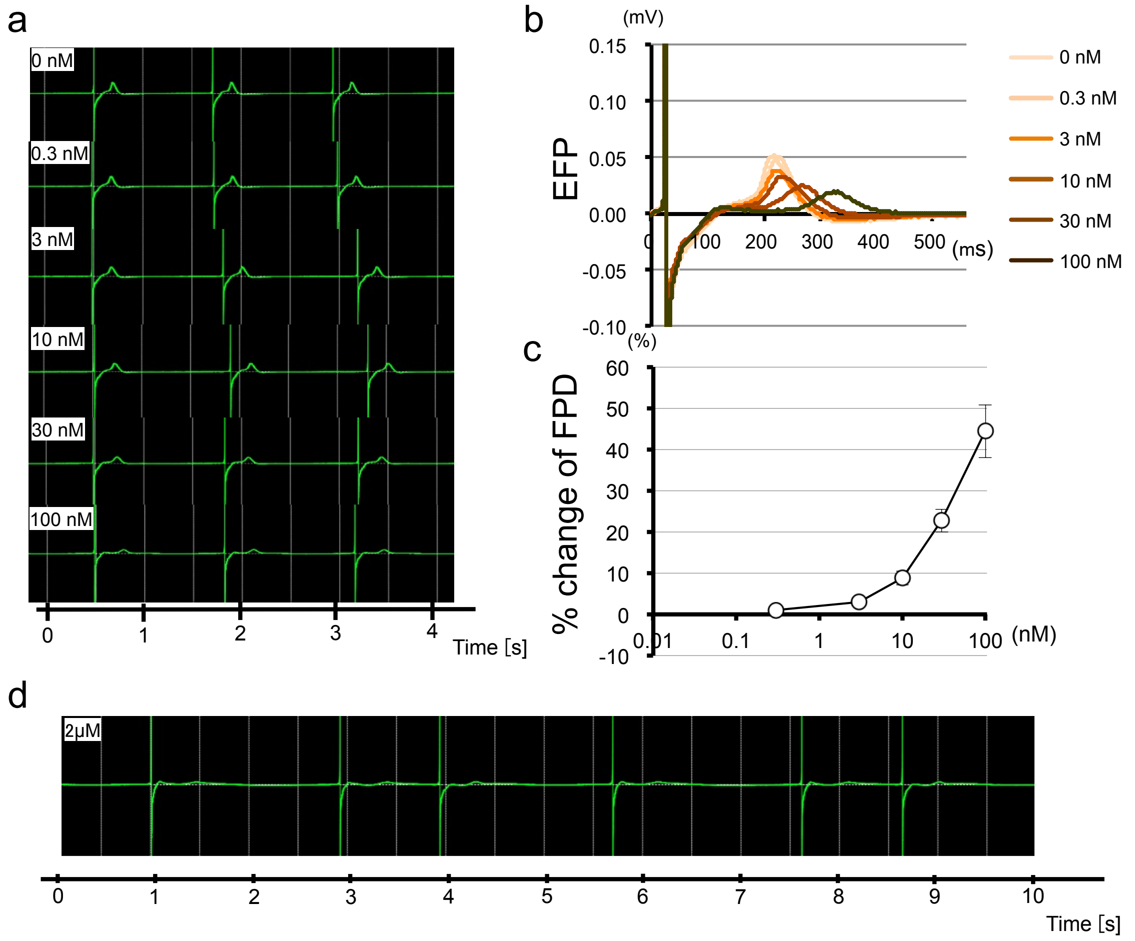

Supplementary Figure 2: Drug-induced EFP changes in cell sheets with 100% cardiomyocytes.

(a) EFP waveforms in response to E-4031 (IKr channel blocker). **(b)** Overlaid EFP waveforms at each E-4031 dosage. **(c)** Relative change in FPD after E-4031 treatment. (±s.d., n=5). **(d)** Representative waveforms in response to a high dose of E-4031 (2 μM).

Supplementary Figure 3

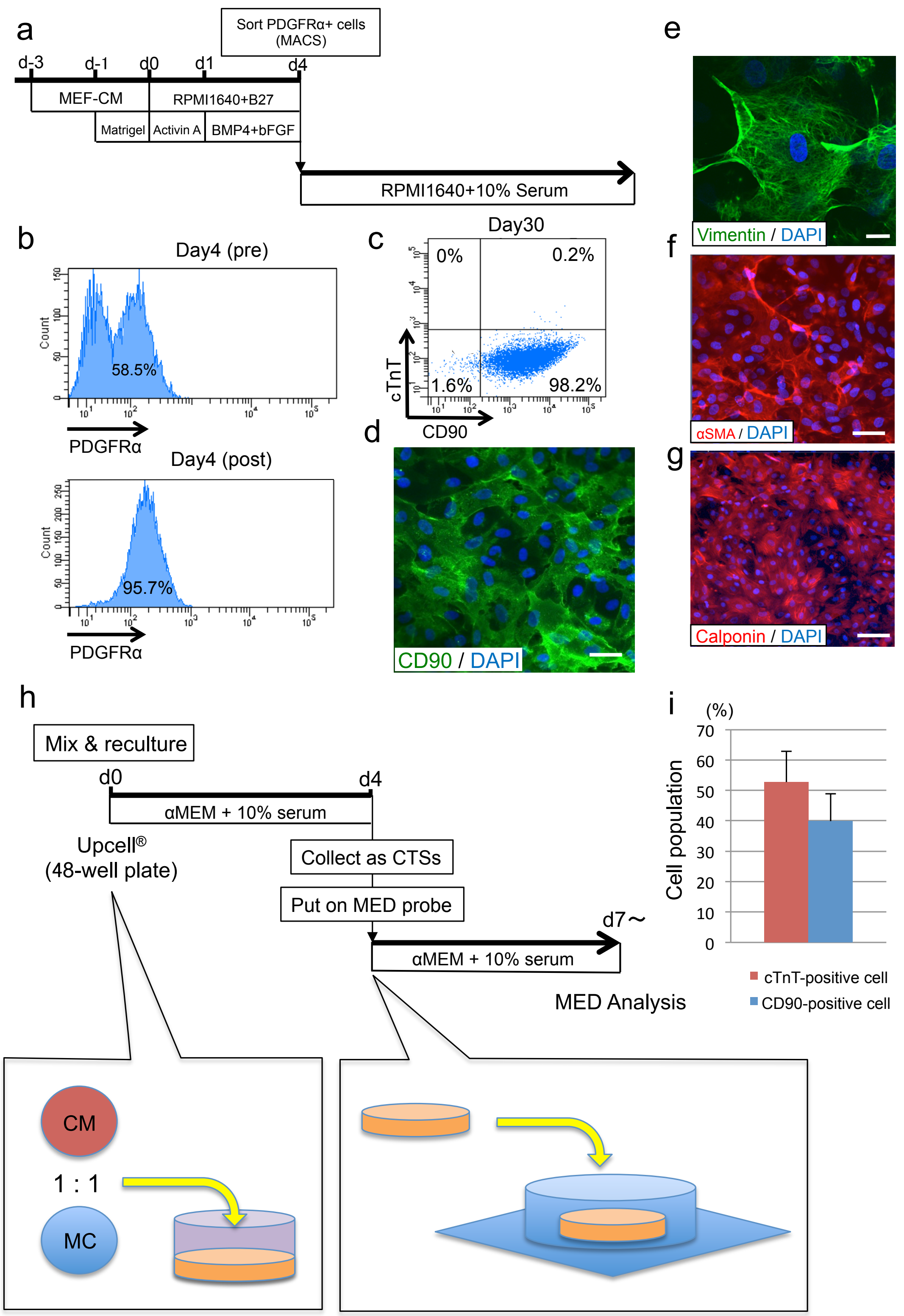

Supplementary Figure 3: Preparation of human iPSC-derived mesenchymal cells

and CTSs consisting of cardiomyocytes and mesenchymal cells.

(a) Schema for the mesenchymal cell differentiation protocol. **(b)** Histograms of the flow cytometry analyses of day 4 PDGFRα-positive cell sorting with MACS. Representative histogram before and after sorting of PDGFRα-positive cells. **(c)** Representative dot plots for CD90 and cTnT on day 30. **(d)** A confocal microscopic image of mesenchymal cells with immunostaining for CD90 (mesenchymal cells, green) and DAPI (nuclei, blue). **(e)** Confocal microscopic images with immunostaining for Vimentin (green) and DAPI (blue). **(f)** Confocal microscopic images with immunostaining for Alpha-smooth muscle actin (αSMA, red) and DAPI (blue). **(g)** Confocal microscopic images with immunostaining for Calponin (red) and DAPI (blue). **(h)** Schema for the preparation of hiPSC-derived 3D CTSs. **(i)** Cellular populations in CTSs analysed by flow cytometry (±s.d., n=4). ActA, activin A; αMEM, alpha minimum essential medium; bFGF, basic fibroblast growth factor; BMP4, bone morphogenetic protein 4; CM, cardiomyocytes; cTnT, cardiac troponin-T; DAPI, 4',6-diamidino-2-phenylindole; MACS, magnetic-activated cell sorter; MCs, mesenchymal cells; MED: multi-electrode device; MEF-CM, mouse embryonic

fibroblast conditioned medium; PDGFRα, platelet-derived growth factor receptor type alpha; SSC, side scatter. Scale bars: 50 μm in **d**, 20 μm in **e**, 50 μm in **f**, and 100 μm in g.

Supplementary Figure 4

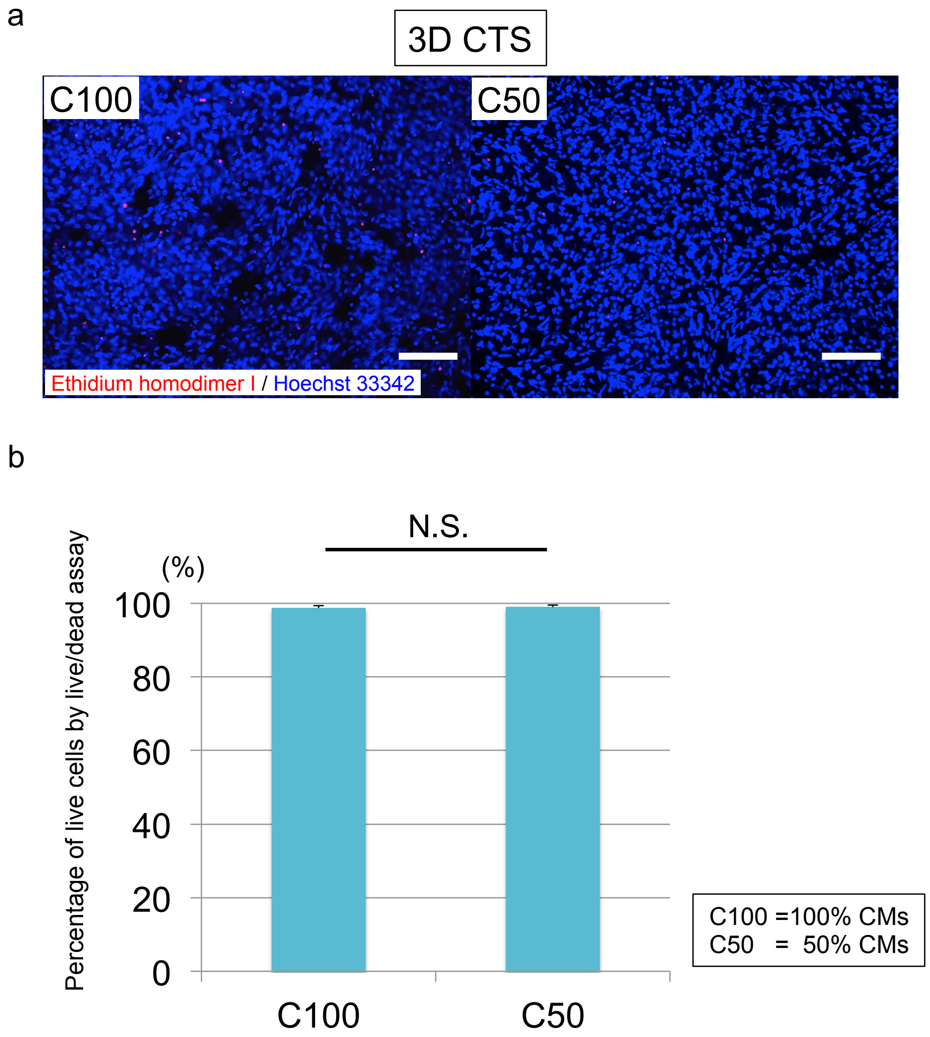

Supplementary Figure 4: Live/Dead assay results.

(a) Representative immunostaining images of the Live/Dead assay in CTSs (C100 / C50). **(b)** Percentage of live cells in CTSs based on the Live/Dead assay (C100 / C50) (±s.d., n=4). C100, sheets with 100% cardiomyocytes. C50, sheets with 50% cardiomyocytes. CMs, cardiomyocytes. Wilcoxon signed-rank test. Scale bars: 100 μm in **a**.

Supplementary Figure 5

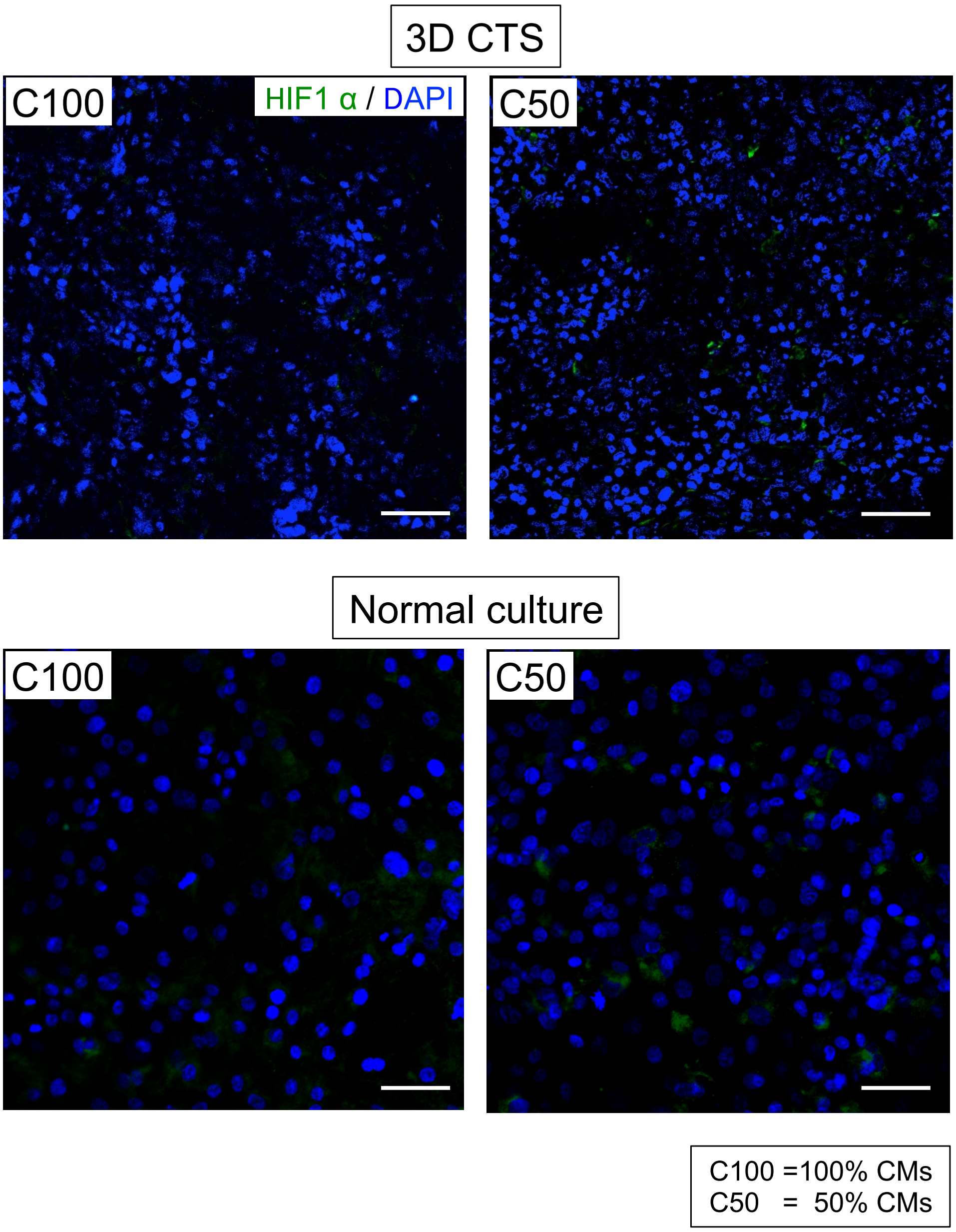

Supplementary Figure 5: HIF1α staining in CTSs.

Representative immunostaining images of HIF1α staining in CTSs (C100 / C50) and normal culture (C100 / C50). C100, sheets or cells with 100% cardiomyocytes. C50, sheets or cells with 50% cardiomyocytes. CMs, cardiomyocytes. Scale bars: 50 μm.

Supplementary Figure 6

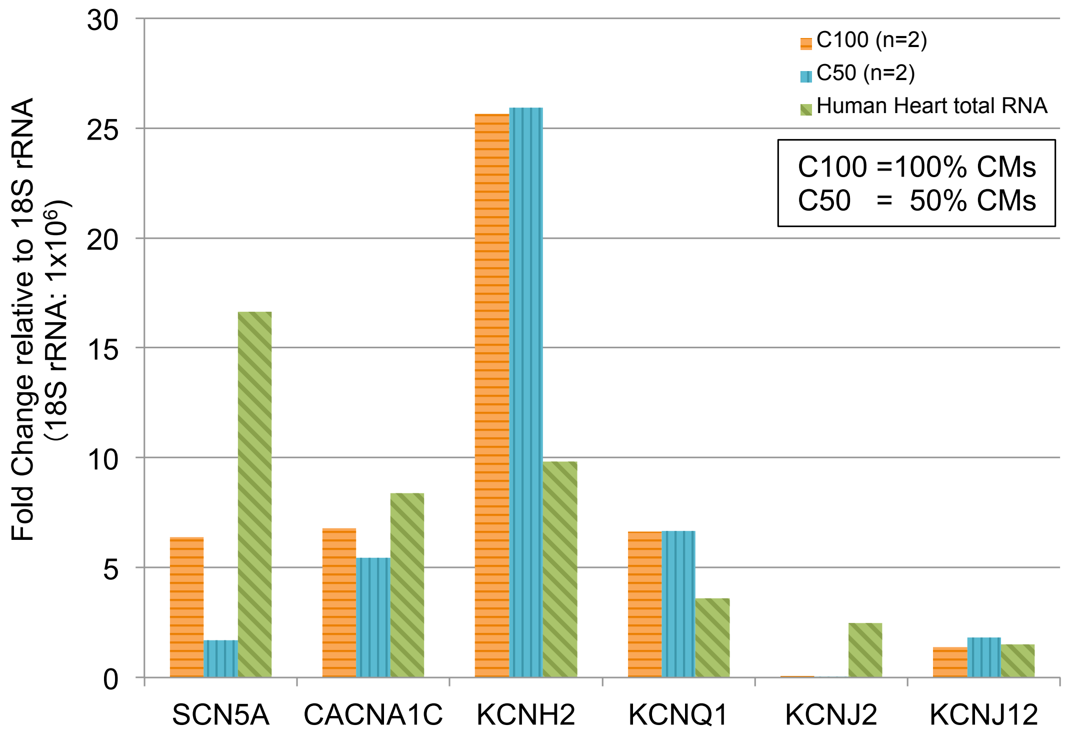

Supplementary Figure 6: RNA extraction and quantitative reverse transcription polymerase chain reaction (RT-PCR).

C100, sheets with 100% cardiomyocytes. C50, sheets with 50% cardiomyocytes. CMs, cardiomyocytes.

Supplementary Figure 7

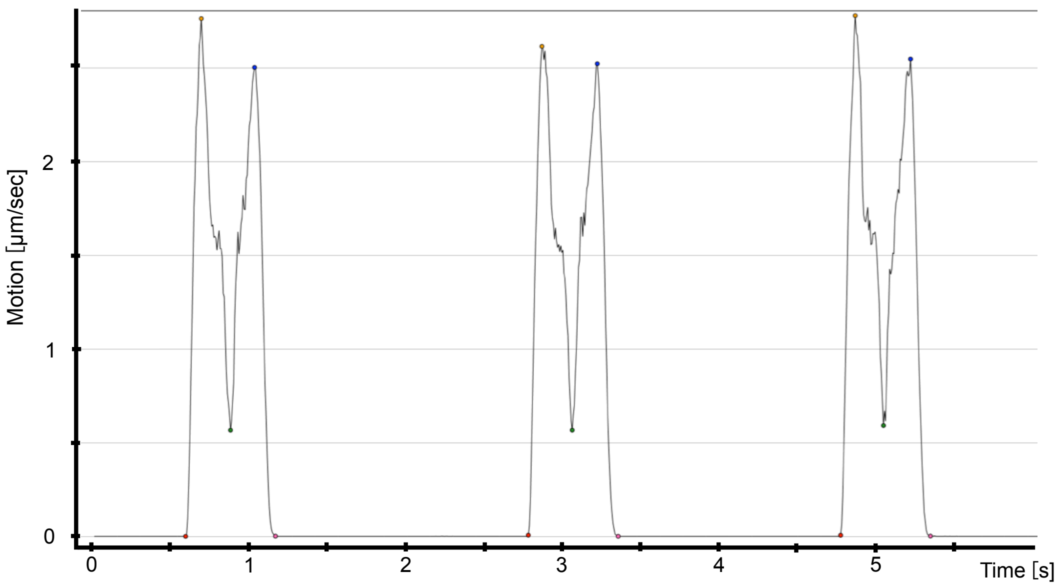

Supplementary Figure 7: Representative waveform of motion vectors in CTSs.

Supplementary Figure 8

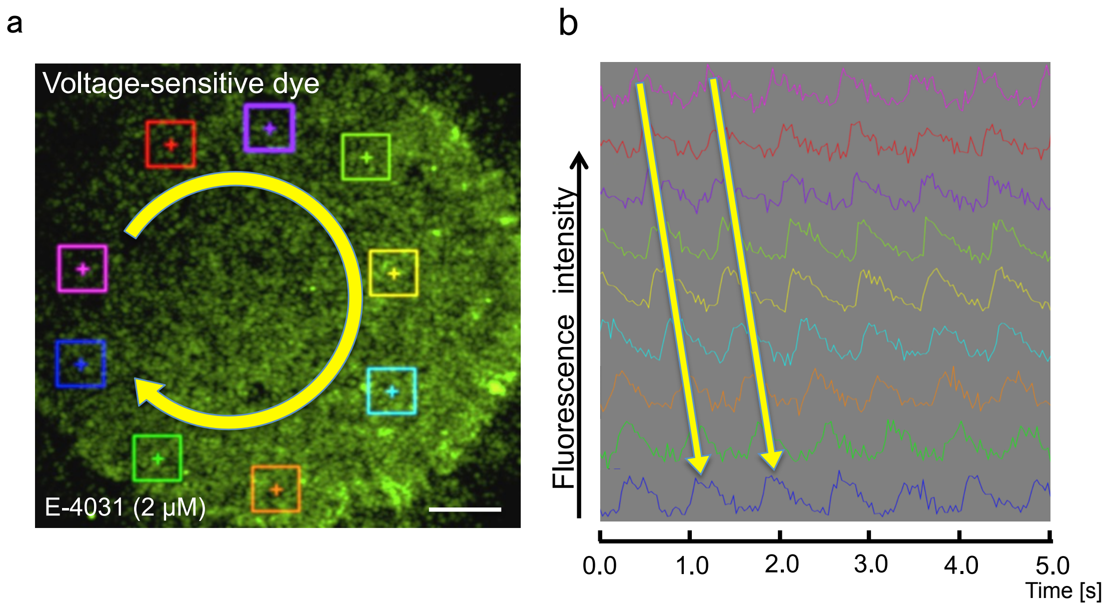

Supplementary Figure 8: Membrane potential oscillation analysis.

(a) A confocal image that includes a representative anchoring spiral wave depicting oscillating membrane potential (E-4031, 2 μM) indicated by FluoVolt, a voltage-sensitive dye (see Supplementary Movie 9). **(b)** Changes in fluorescence intensity. The intensity was measured at 9 points in the sheet (ROIs are shown in **a**). ROI, region of interest. Scale bar: 1 mm.

Supplementary Figure 9

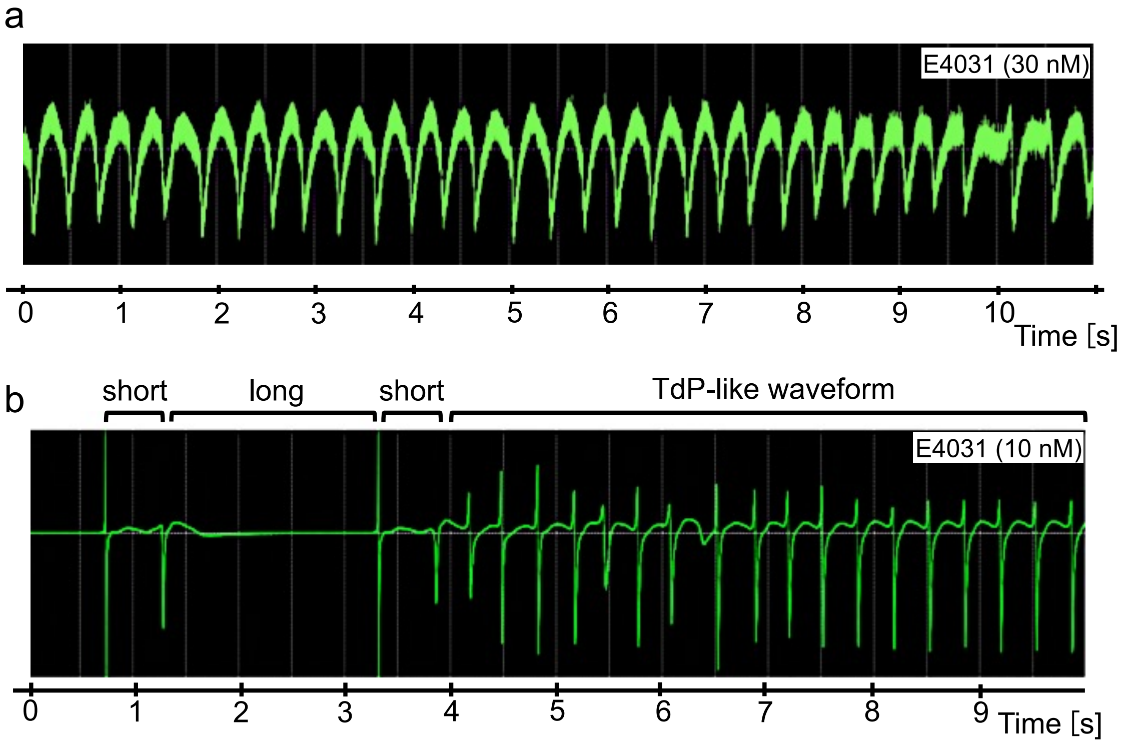

Supplementary Figure 9: Reproduction of drug-induced TdP-like waveforms in CTSs that included MiraCell® cardiomyocytes.

(a) TdP-like waveform induction by E-4031 (30 nM) in CTSs that included MiraCell® cardiomyocytes. **(b)** Representative short-long-short sequence in the initiation of TdP-like waveform by E-4031 (10 nM) in CTSs that included MiraCell® cardiomyocytes.

Supplementary Figure 10

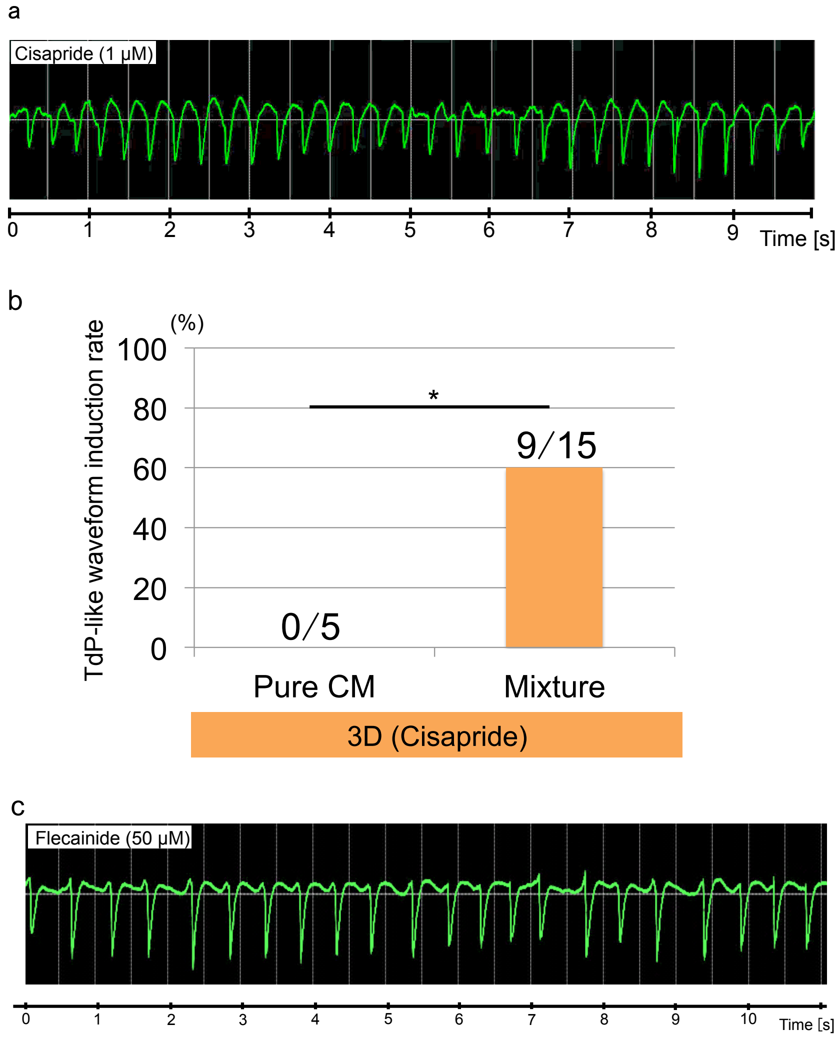

Supplementary Figure 10: Reproduction of drug-induced TdP-like waveforms in CTSs.

(a) Representative polymorphic ventricular tachycardia TdP-like waveform induced by cisapride (1 μM). **(b)** TdP-like waveform induction rates by cisapride. CM, cardiomyocytes. Wilcoxon signed-rank test *$P<0.05$. **(c)** Representative polymorphic ventricular tachycardia TdP-like waveforms induced by flecainide (50 μM).

Supplementary Figure 11

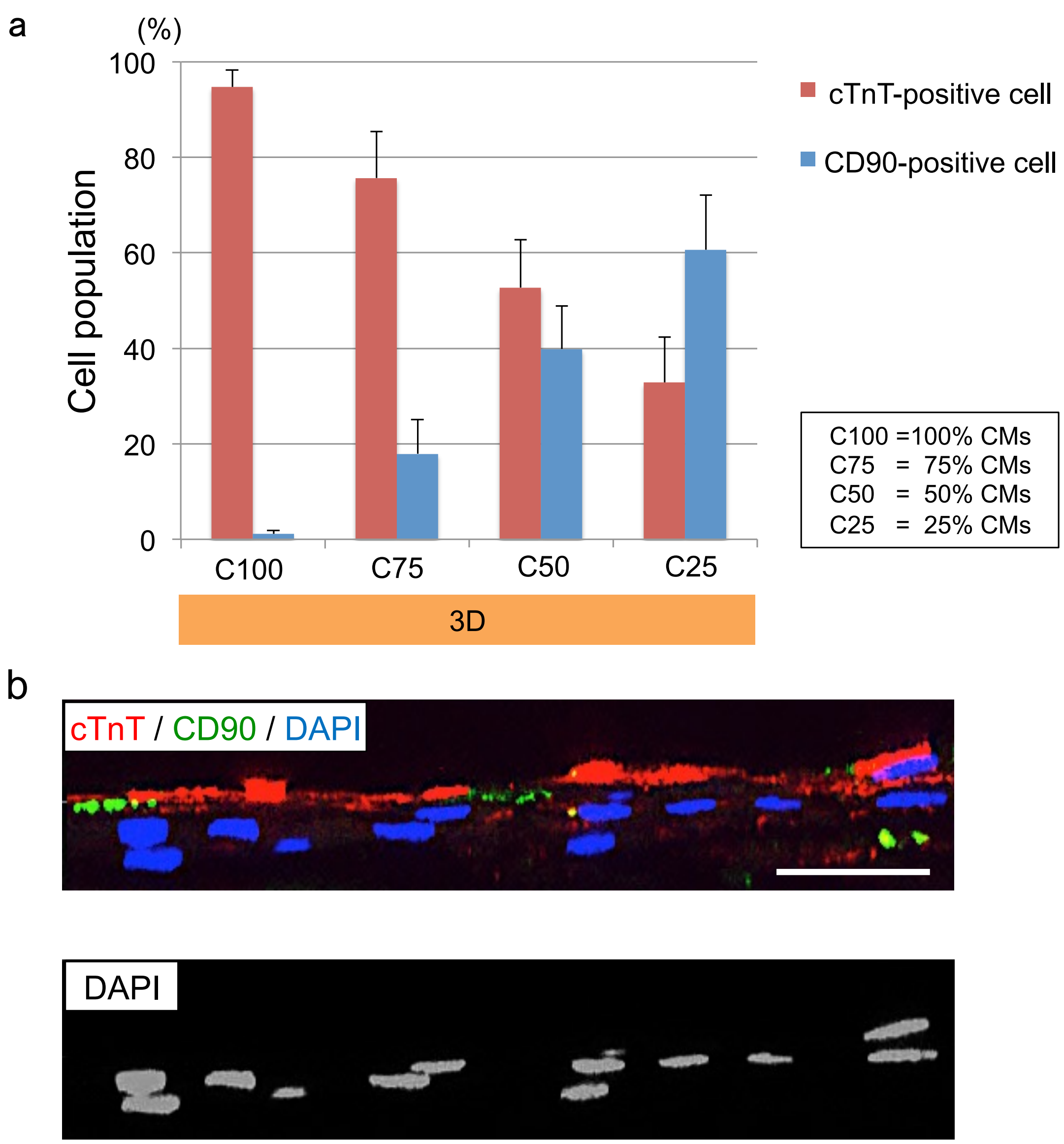

Supplementary Figure 11: Cellular composition of 3D CTSs and 2D culture.

(a) Cellular compositions of 3D CTSs, as analysed by flow cytometry (±s.d., n=4). C100 (generated with 100% cardiomyocytes) sheets consisted of 94.7±3.6% CMs and 1.2±0.7% MCs. C75 (generated with 75% cardiomyocytes) sheets consisted of

75.7±9.8% CMs and 17.9±7.2% MCs. C50 (generated with 50% cardiomyocytes) sheets consisted of 52.7±10.0% CMs and 39.9±9.0% MCs. C25 (generated with 25% cardiomyocytes) sheets consisted of 32.8±9.5% CMs and 60.7±11.5% MCs. CMs, cardiomyocytes. MCs, mesenchymal cells. **(b)** Confocal microscopic images of a vertical section of an immunostained 2D culture with 50% cardiomyocytes. cTnT (red), CD90 (green), and DAPI (top: blue; bottom: white). Scale bars: 25 μm.

Supplementary Figure 12

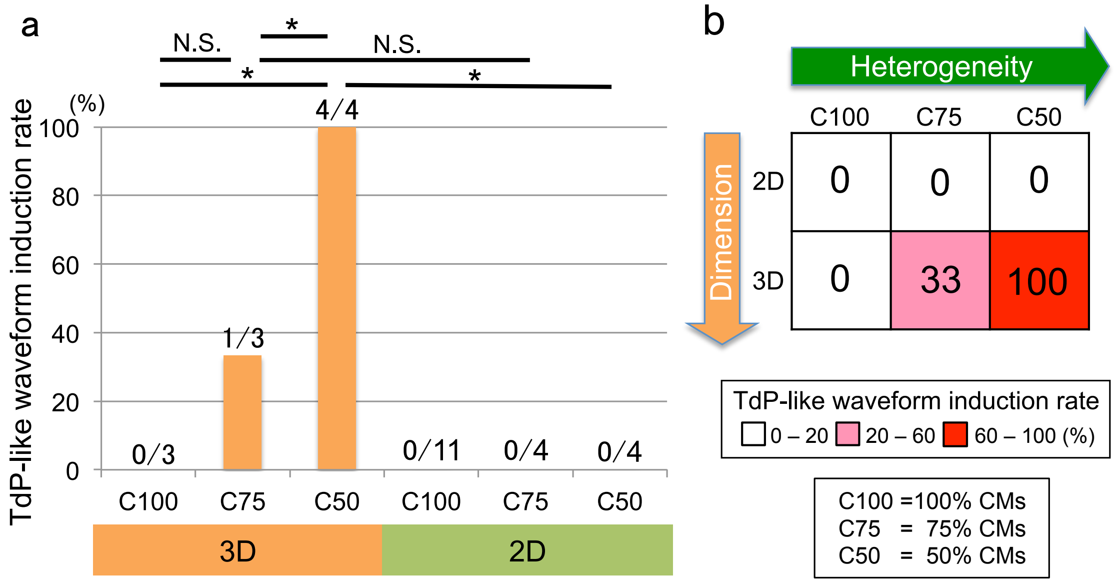

Supplementary Figure 12: Induction rates of TdP-like waveforms in CTSs that include MiraCell® cardiomyocytes.

(a) Induction rates of TdP-like waveform in CTSs of different heterogeneities and dimensions. C100, cells or sheets with 100% cardiomyocytes. C75, cells or sheets with 75% cardiomyocytes. C50, cells or sheets with 50% cardiomyocytes. CMs, cardiomyocytes. Fisher's exact test *P<0.05. **(b)** Relationship of TdP-like waveform incidence with cell heterogeneity and dimension.

Supplementary Figure 13

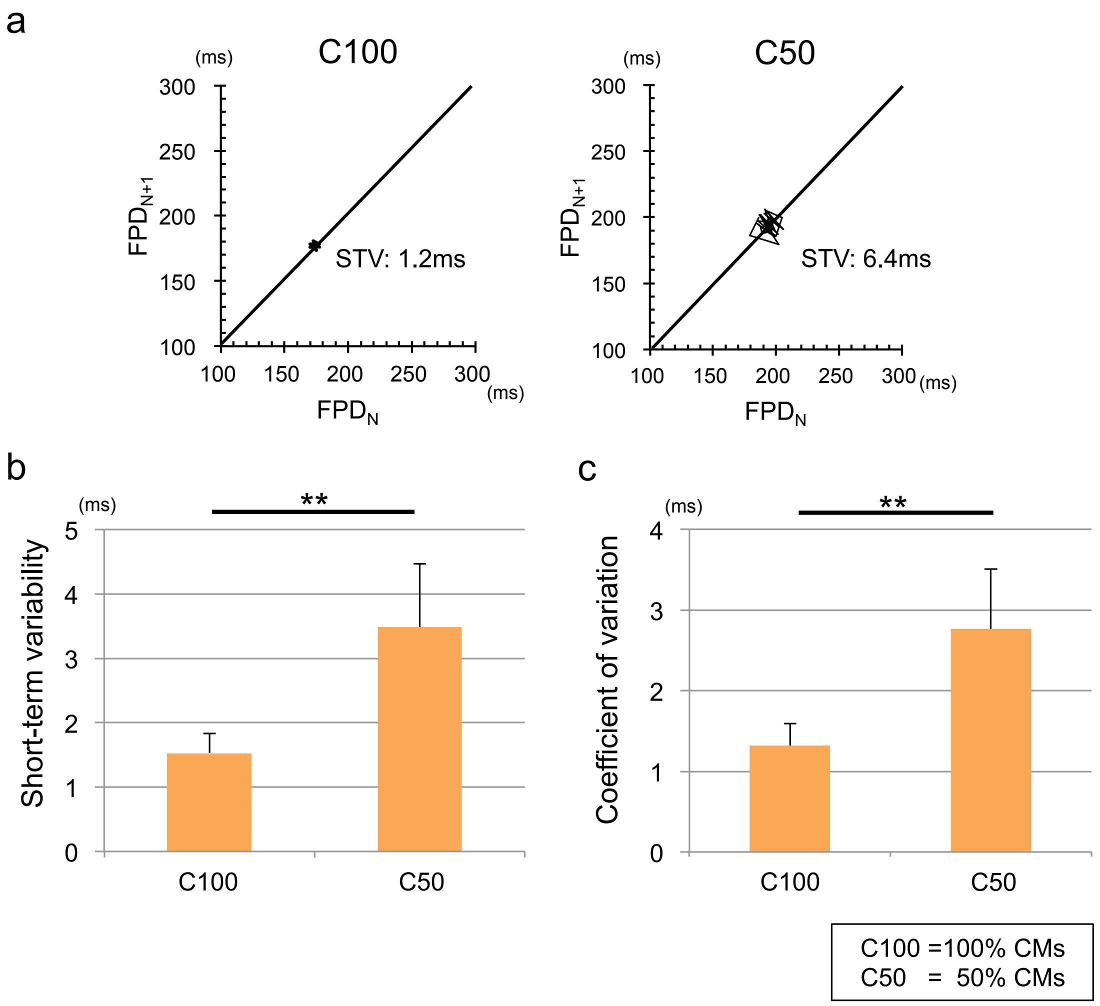

Supplementary Figure 13: Beat-to-beat variability of the repolarization in CTSs.

(a) Representative Poincaré plots of the FPD intervals in CTSs (C100 / C50). STV, short-term variability. **(b)** Histogram of STV of repolarization of FPD intervals in CTSs. **(c)** Histogram of the coefficient of variation of FPD intervals in CTSs. C100, sheets with 100% cardiomyocytes (±s.d., n=9). C50, sheets with 50% cardiomyocytes (±s.d., n=6). CMs, cardiomyocytes. Wilcoxon signed-rank test *P<0.05 and **P<0.01.

Supplementary Figure 14

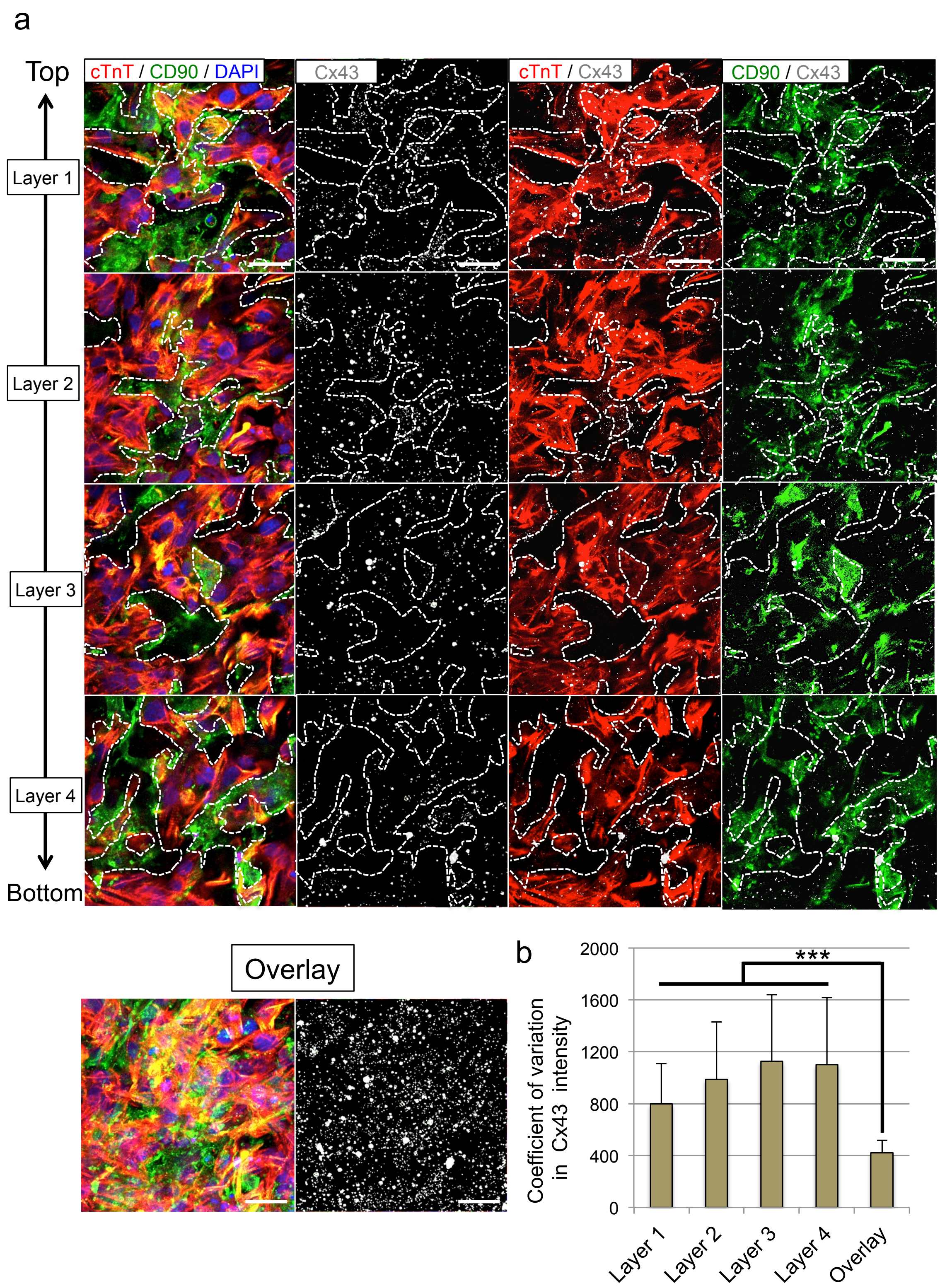

Supplementary Figure 14: Immunostaining of Cx43 in serial layers of CTSs.

(a) Representative Cx43 immunostaining in serial layers of CTSs with 50% cardiomyocytes. White dotted lines show the border between the cardiomyocyte area and mesenchymal cell area. Scale bars: 25 μm. **(b)** Coefficient of variation in Cx43 intensity. (±s.d., n=240 lines). Steel's test ***P<0.001.
