# Supplementary material for: Modelling Torsade de Pointes arrhythmias in vitro in 3D human iPS cell-engineered heart tissue
Source: Nat Commun. 2017 Oct 20;8:1078. doi: 10.1038/s41467-017-01125-y (PMC5715012; doi:10.1038/s41467-017-01125-y)
Supplement: Supplementary file 3 — Description of Additional Supplementary Files [file 41467_2017_1125_MOESM3_ESM.pdf]

Supplementary Movie legends:

Supplementary Movie 1: Continuous horizontal cross-sectional confocal images of 3D Cardiac Tissue Sheets (CTSs) from top to bottom. These images were immunostained for cTnT (cardiomyocytes, red), CD90 (mesenchymal cells, green), and DAPI (cell nuclei, blue). Scale bar: 25 μm. cTnT, cardiac troponin-T; DAPI, 4',6-diamidino-2-phenylindole.

Supplementary Movie 2: Extracellular field potential (EFP) of representative sustained tachyarrhythmia recorded with a multi-electrode device. Upper movie: monomorphic ventricular tachycardia-like waveforms related to Fig. 2e (E-4031, 1 μM). Lower movie: Torsade de Points (TdP)-like waveforms related to Fig. 2f (E-4031, 1 μM).

Supplementary Movie 3: Representative unidirectional biphasic wave propagation and waveform before E-4031 treatment in Motion Vector Prediction (MVP) and EFP. Scale bar: 1 mm.

Supplementary Movie 4: Representative anchoring spiral wave reentry in MVP and monomorphic ventricular tachycardia-like waveforms in EFP after E-4031 treatment (1 μM). Scale bar: 1 mm.

Supplementary Movie 5: Transition from unidirectional biphasic wave propagation to anchoring spiral wave re-entry in MVP and monomorphic ventricular tachycardia-like

waveforms in EFP (simultaneous recording of EFP and cell motion) after E-4031 treatment (2 μM). The transition occurs at 14 s from the start of the movie. At first unidirectional wave propagations are observed from different origins, but then, in some cases, interfere with each other to induce spiral wave re-entry. Scale bar: 1 mm.

Supplementary Movie 6: Representative meandering of spiral wave center in MVP and TdP-like waveforms in EFP, related to Fig. 3e (E-4031, 1 μM). Left movie shows signals obtained with a higher threshold to clearly visualize the clockwise spiral wave re-entry of the whole CTS. Right movie shows signals obtained with a lower threshold to clearly visualize meandering of the spiral wave center. The center moves around clockwise. Scale bars: 1 mm.

Supplementary Movie 7: Representative meandering (drifting) of spiral wave center in MVP and TdP-like waveforms in EFP, related to Fig. 3f (E-4031, 2 μM). Left movie shows signals obtained with a higher threshold to clearly visualize the counterclockwise spiral wave re-entry of the whole CTS. Right movie shows signals obtained with a lower threshold to clearly visualize meandering of the spiral wave center. Drifting of the center occurs at 8 sec (toward left), 12 sec (toward left), 24 sec (toward bottom), 28 sec (toward left), 46 sec (toward left) and 52 sec (toward left) in the movie. Scale bars: 1 mm.

Supplementary Movie 8: Tracking of representative anchoring (left) (E-4031, 1 μM) and meandering (right) (E-4031, 2 μM) of spiral wave re-entry in MVP, related to Fig. 3f. The white circles show the center of spiral wave, and the black lines show the trace of the center.

Representative EFPs for each MVP are shown below. Scale bars: 1 mm.

Supplementary Movie 9: Representative anchoring spiral wave re-entry in membrane potential oscillation after E-4031 treatment (2 μM). The spiral wave can be observed clockwise. Scale bar: 1 mm.

Supplementary Movie 10: Representative meandering of spiral wave center in MVP and TdP-like waveforms in EFP using MiraCell®, related to Supplementary Fig. 9 (E-4031, 30 nM). Left movie shows signals obtained with a higher threshold to clearly visualize the clockwise spiral wave re-entry of the whole CTS. Right movie shows signals obtained with a lower threshold to clearly visualize meandering of the spiral wave center. Scale bars: 1 mm.

Supplementary Movie 11: Representative wavefront and wavetail in spiral wave re-entry in MVP. The spiral wave can be observed counterclockwise. Scale bar: 1 mm.
